# Hardware Selection and Performance of Low-Cost Fluorometers

**DOI:** 10.3390/s22062319

**Published:** 2022-03-17

**Authors:** Jase L. Hixson, Adam S. Ward

**Affiliations:** O’Neill School of Public and Environmental Affairs, Indiana University, Bloomington, IN 47405, USA; adamward@indiana.edu

**Keywords:** fluorometer, low-cost, DIY sensors, uranine, turbidity sensitivity, temperature sensitivity

## Abstract

Access to and extensive use of fluorometric analyses is limited, despite its extensive utility in environmental transport and fate. Wide-spread application of fluorescent tracers has been limited by the prohibitive costs of research-grade equipment and logistical constraints of sampling, due to the need for high spatial resolutions and access to remote locations over long timescales. Recently, low-cost alternatives to research-grade equipment have been found to produce comparable data at a small fraction of the price for commercial equipment. Here, we prototyped and benchmarked performance of a variety of fluorometer components against commercial units, including performance as a function of tracer concentration, turbidity, and temperature, all of which are known to impact fluorometer performance. While component performance was found to be comparable to the commercial units tested, the best configuration tested obtained a functional resolution of 0.1 ppb, a working concentration range of 0.1 to >300 ppb, and a cost of USD 59.13.

## 1. Introduction

Fluorescent tracers are an important tool in our study of environmental transport and fate in streams and rivers [1,2,3,4,5,6]. For example, fluorescent tracers are used to study travel times, estimate the persistence of exposure or estimate risk after a spill, e.g., [7], and estimate transport processes and coefficients, e.g., [8]. The environmental fate of reactive fluorescent tracers is directly related to conditions they experience during transport, including solar irradiance, hyporheic storage, pH, temperature, and turbidity [9,10,11]. Although information from tracer studies is valuable to our understanding of transport and fate, the high cost of research-grade field equipment limits the use of fluorescent tracers. While grab samples and laboratory analyses do offer a lower-cost surrogate, that strategy is expensive in person-hours and analytical costs. Moreover, the desire for high fidelity in both space and time, including in remote locations and during adverse conditions where fieldwork is unsafe, mean sensors must be rugged, robust, and able to operate as stand-alone platforms for extended deployments.

Recent advancements in microcontrollers and microprocessors have ushered in a new wave of low-cost equipment for environmental sensing [7,12,13,14,15,16,17,18,19,20]. For example, commercial in situ chlorophyll a fluorometers cost more than USD 3000 while low-cost alternatives with comparable performance can be produced for less than 5% of that cost [16]. Development of low-cost alternatives is not without challenge. Sensor performance is known to vary in response to the consistency of components, recording bitrate, and the design and assembly of components themselves, e.g., [21]. However, these obstacles can be overcome through careful calibration. In one recent example, low-cost turbidimeters produced for around 4% of the cost for a commercial alternative were documented to achieve performance with r^2^ = 0.9996 with commercial equipment [15].

Commercial equipment for fluorescent solute tracer studies is precise, accurate, rugged (field deployable models), and expensive (Table 1). Early work with solute tracers focused on collection of field samples with subsequent laboratory analysis, or the use of portable generators in the field [22,23,24]. Since the introduction of fluorometers, routine innovation has spurred advances in equipment performance and robustness, while reducing equipment costs [16,18,25,26,27,28]. While advances have occurred through the use of varying methodologies and components [25,26,29,30], more recent advancements have integrated 3D printed components to construct niche components for specific components and research objectives [14,28,31,32,33].

Modern laboratory fluorometers range in cost from USD 6000 to USD 39,000 with detection limits ranging from 0.01 to 2.0 ppb for common tracers like uranine (Table 1). Since the early 1990s, field deployable fluorometers have been commercially available. Current options range in cost from USD 1000 to USD 8000 with comparable performance to laboratory equipment (0.01–10.0 ppb; Table 1). While this equipment is accurate and reliable, the price-point limits access to this equipment and, consequently, the scientific understanding that could be gained by their use.

The goal of this study is to prototype and document performance of a low-cost fluorometer for uranine built from readily available components. Here, we explore alternative hardware combinations, benchmarking performance against a commercial, field-deployable fluorometer [34,35]. We assess performance across ranges of concentrations, turbidity, and temperature to characterize drift and corrections, following the procedures used to assess performance and uncertainty in commercial units [10]. Ultimately, this study details the cost, construction, and performance of a low-cost fluorometer that can be readily adapted to a range of applications and tracers.

## 2. Materials and Methods

### 2.1. Fluorometer Design and Components Tested

Our prototype is designed around the Adafruit Feather m0 Adalogger and DS3231 Precision Real Time Clock mounted together on the FeatherWing Doubler to add structural support. Based around this platform, we tested configurations including varied logger bit resolution, LED excitation sources, emission sensors, and optical filters (Table 2). Programming was based on modifying component-specific example codes published by the manufacturer to ensure performance could be achieved with minimal coding background, and sample code is included, along with wiring schematics and product links, in the data associated with this study [36].

We tested loggers using native 8- and 10-bit resolution on the m0 board and added configurations with an additional ADS1115 16-bit resolution converter after preliminary screening of hardware alternatives. Across all designs, we used a Broadcom Limited 470 nm (460–480 nm) and 12,000 mcd LED to provide excitation for uranine, and a Kingbright 630 nm (605–655), 12,000 mcd LED to sense turbidity. Both LED wavelengths were selected to closely match the commercially available GGUN FL30 [35]. Importantly, the wavelength for turbidity is outside the range of excitation/emission spectra for common solute tracers including uranine, rhodamine WT, resazurin, and resorufin, enabling future expansion of this design for other tracers without requirement to change the turbidity sensor. LEDs were mounted using the IO Rodeo Colorimeter LED Board Ver. B to ensure each light source had the same angle of incidence to the optical cell. In addition to the optical sensors, a 10 kΩ Precision Epoxy Thermistor (resolution 0.25 °C) was placed directly in the water column to record water temperature. This placement differs for the placement of a temperature probe on the GGUN FL30, where the thermistor is inside the waterproof housing but isolated from the water by the Pyrex flow-through tube [37].

Low-cost photocells or photoresistors may not be accurate for measuring exact light intensity, instead recording variable values even at a fixed intensity [21]. To increase the reliability of designs using photoresistors (configurations A–C, H), we collected a large number of replicate observations samples for each case with the goal of averaging measurements to produce a functional 2 s sampling rate to improve precision. We evaluated performance of the 8-bit and 10-bit sensors by averaging (or ‘stacking’) 40,000 replicate measurements collected in about 1 s. The 16-bit sensor data were based on averaging 1000 samples collected within about 1 s, with the lower number compared to 8- and 10-bit owing to the computational overhead in using the 16-bit converter. Additionally, instability in optical instruments has been documented prior to light sources being ‘warmed up’ [16]. Therefore, to remove this instability, the LEDs were activated for 1 s prior to the start of measurements, yielding a functional 2 s temporal resolution for each configuration tested.

### 2.2. Experiment 1: Benchmarking Low-Cost Fluorometer to Commercial Unit Performance

**Figure 1:**
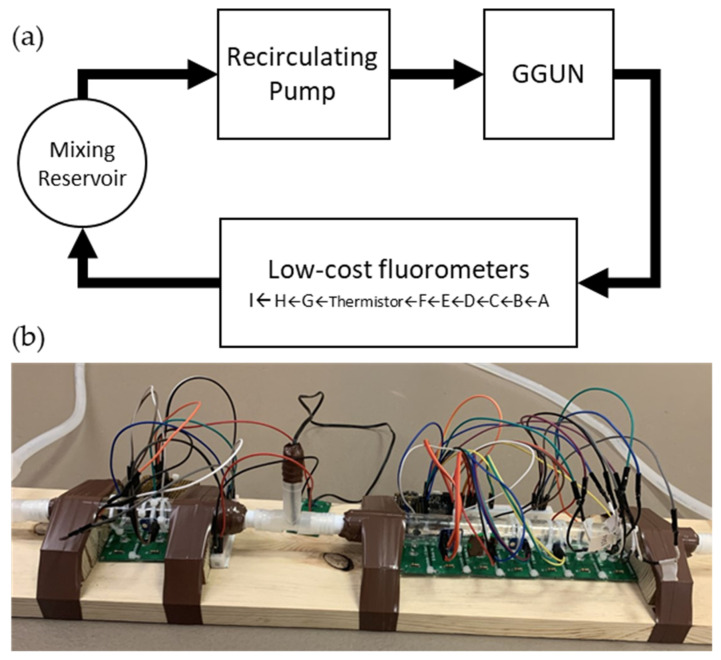
(**a**) Experimental setup of recirculating system to test fluorometer designs and benchmark performance with a commercial GGUN FI30. (**b**) Initial layout of low cost fluorometer designs for simultaneous testing. LEDs and sensors were oriented at 90-degree angles to prevent detecting the light source over fluorescence. All experiments occurred with the fluorometers being placed in a dark container to prevent ambient light from being measured. Flow cells were connected via PVC barbed couplers and silicone tubing fitted over the flow cells and taped to prevent leaks.

Experiments were performed using a recirculating system consisting of a GGUN FL30 field fluorometer, two optical cells (Pyrex tubes) with an outside diameter of 20 mm, and a constantly mixing reservoir operated in series (Figure 1). The system was plumbed with silicone tubing, with a total volume of 500 mL circulating through the system. The system was initially filled with 0 ppb MilliQ water to establish background readings for each sensor. Known masses of uranine were added to the mixing reservoir to incrementally increase concentrations across the range of 0 to 300 ppb uranine, matching the typical calibration range used for the commercial unit [37]. Initial increases in concentration were small (1.5 ppb) to assess low concentration instrument sensitivity. Uranine concentrations between 10–100 ppb were increased in 10 ppb steps, and for concentrations above 100 ppb we increased concentration in 100 ppb steps up to 300 ppb. At concentrations above 100 ppb, our goal was to determine if the instruments would experience saturation at high concentrations. Concentrations were allowed to stabilize for at least 3 times the residence time in the recirculating system before observations were made for calibration to ensure uranine was well mixed. In all cases, a regression between the GGUN and low-cost configurations was used to assess performance, with a linear correlation coefficient of 1.0 representing perfect performance.

### 2.3. Experiment 2: Assessment of the Functional Resolution and Sensitivity of Low-Cost Sensors

To assess the ability of the low-cost sensors to resolve fine changes in concentration, measurements from the low-cost sensors and GGUN were compared by measuring small changes in concentration in the operation range of 0.99 to 111 ppb. We achieved a slow change in concentration by allowing the system to recirculate while photolysis slowly degraded the uranine. The recirculating system was filled with a concentration of 111 ppb of uranine and run continuously until concentration approached 0 ppb after 133 h. After the first 4000 min, we added an external light source to increase the photolysis rate and accelerate the experiment. Each hardware configuration collected one observation approximately every 10 s during the study, with one reading at a time to ensure no interference would occur between sensors. The calibration curves from Experiment 1 were used to convert raw mV readings to uranine concentrations. These concentrations were compared to GGUN concentrations to assess performance based on goodness of fit (r^2^) between low-cost designs and commercial alternatives. For each 22 min interval, we tabulated the mean, median, and standard deviation of the measurements collected. To assess the uncertainty of individual measurements we calculated the 95% confidence interval for each concentration tested as two times the standard deviation (σ) divided by the mean (μ). Comparable data are not available for the commercial GGUN as the unit already stacks measurements and reports only the averaged behavior, but reported uncertainty is 0.02 ppb (Table 1).

### 2.4. Experiment 3: Robustness of Performance with Temperature and Turbidity

Measurements from fluorometers are known to be susceptible to several environmental factors including temperature, turbidity, pH, and background fluorescence of organic matter [10,38,39,40]. Here, we follow established procedures for assessing performance as a function of temperature and turbidity [10]. Briefly, the recirculating system was initially filled with room temperature stock concentrations of uranine (6, 20, 70 ppb concentrations were each tested independently). The entire system was placed in a refrigerator and cooled to about 4 °C, then removed from the refrigerator and allowed to return to room temperature. Changes in intensity of fluorescence were calculated as the percent change from the initial, room-temperature signal. In addition to the GGUN FL30 used in these experiments, temperature and turbidity sensitivity of an additional GGUN FL30 was determined according to Blaen et al., [10] to determine the range of sensitivities across several commercial units. Performance as a function of turbidity was assessed by adding a known volume of 1000 NTU solution (Hach StablCal 1000 NTU Turbidity Standard) to increase turbidity in increments of 10 NTU over the range of 0 to 60 NTU. These experiments were repeated for uranine concentrations of 6, 50, and 80 ppb. As in experiment 1, the system was allowed to circulate to achieve equilibrium prior to collecting observations at each turbidity value tested, with at least three residence times of the system allowed to pass between observations. Based on the results of Experiments 1 and 2, we only tested temperature and turbidity sensitivity for hardware configurations A, B, F, G, and H.

## 3. Results

### 3.1. Low-Cost Fluorometer Performance

#### 3.1.1. Comparison of Components for Low-Cost Fluorometers (Bit Rate and Sampling Frequency)

**Figure 2:**
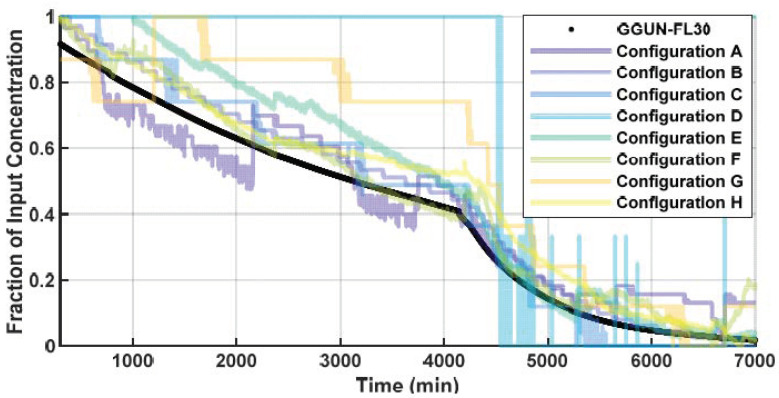
Concentration profile used to calibrate and determine resolution of low-cost sensors. Each sensor, with the exception of configuration D, was able to accurately measure changes in uranine concentrations. Configurations with ‘stair step’ pattern still achieved a high r^2^, but were less sensitive to changes in concentration.

Configurations with higher bit resolution resulted in higher r^2^ values with known concentrations. For example, the same light source and detector showed significant improvement when increasing from 8 bit (r^2^ = 0.37; configuration I) to 16 bit (r^2^ = 0.92; configuration F) due to the increased precision of each measurement. This improvement is realized because the standard 8-bit pins divide the 3.3 V delivered by the Adalogger into 256 values (i.e., 28), which results in a resolution of 12.9 mV. For comparison, the 10-bit divides the range into 1024 values (210; 3.22 mV resolution) and the 16-bit sensors into 65,536 values (216; 0.05 mV resolution) and is visible as ‘stair steps’ in the experimental results (Figure 2).

Increasing bit rate, while increasing the mV resolution, did not increase performance across all sensors. The equipment showing greatest improvement with increasing bit resolution are from components with minimal noise, such as the sensor in configuration F that has a true log-linear relationship with fluorescence. However, the photoresistors had diminished improvement beyond the on board 10-bit resolution due to the inherent noise in these sensors. Uncertainty in the measurements can be overcome with increased sampling and averaging of measurements. In order to achieve 2 s sample resolution, a greater number of samples could be collected per second for 8-bit (r^2^= 0.35–0.99 @ 40,000 samples averaged per measurement; configurations C, D, G, I) and 10-bit (r^2^ = 0.95–1.0 @ 40,000 samples averaged per measurement; configurations A, B) resolution, which resulted in increased precision. The additional resolution of the 16-bit (r^2^ = 0.92–0.99 @ 1000 samples averaged per measurement; configurations E, F, H) did improve performance evaluated as r^2^ with the commercial unit, but reduced the ability to ‘stack’ or average replicate measurements, leaving the device more vulnerable to error associated with component noise. The addition of a uranine-specific optical filter allowed for better detection of uranine (r^2^ of 0.37 and 0.85 on 8- and 16-bit configurations, respectively; configurations I and G). While the optical filter did improve performance, components A–C, E, F, and H achieved a higher r^2^ for less than 10% of the cost of the optical filter alone, suggesting its cost may not be justified by its benefit in this application.

#### 3.1.2. Performance Comparison of Low-Cost vs. Commercial (Comparison to GGUN)

Limited resolution of lower bitrate sensors inherently constrains instrument performance at low concentrations, while still allowing greater resistance to saturation at higher concentrations (Figure 2). While the 8-bit sensors were found to have an r^2^ up to 0.99, they could only resolve concentration changes of 12 ppb, resulting in frequent jumps or steps in the estimation of concentrations (Figure 2). The highest r^2^ for the low-cost designs was achieved using configuration B. To gain a higher sensitivity than produced in the 8-bit components, the 10-bit and 16-bit components were used in subsequent trials (e.g., configurations B and H). Resolution of the 10-bit sensor was 1.2 ppb, while the 16-bit sensor was 0.1 ppb and achieved an r^2^ of >0.999 and 0.99. Configurations D and I produced overall low-quality data at 8-bit resolution (r^2^ of 0.35 and 0.37, respectively). However, the same detector as F but with a higher 16-bit resolution was able to produce an r^2^ of 0.92. Similarly, measurement uncertainty generally decreased as bit rate increased, indicating more repeatable measurements (Table 2).

**Figure 3:**
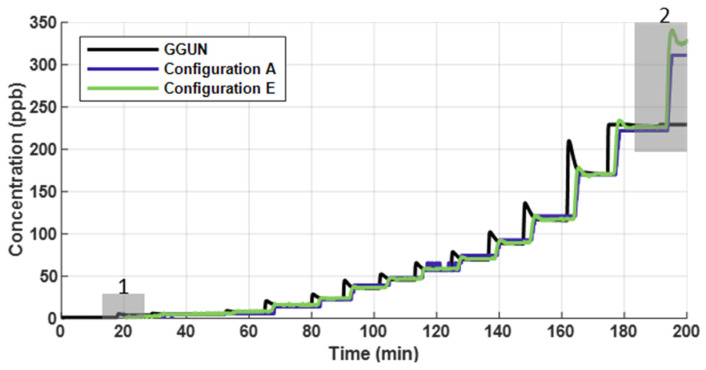
Concentration profiles that represent the commercial unit, 16-bit sensor (Configuration E), and 10-bit sensor (Configuration A). The 10-bit sensor had a lower resolution and did not detect the initial change in concentration (grey box 1). The commercial unit was unable to detect changes in concentrations at >200 ppb due to saturation (grey box 2). Only at the highest and lowest concentrations tested did the low-cost and commercial fluorometers diverge in their ability to accurately measure uranine concentrations.

Across an operating range of 1 to 305 ppb uranine, low-cost fluorometer performance was comparable to that of a commercial instrument (Figure 3). Percent error for each step in concentration was calculated after each step equalized in concentration, comparing the calibrated fluorometer concentrations to the known concentrations. Average percent error for the commercial unit was 11.2%, while Configuration A and E had an average error of 13.9% and 10.3%, respectively. Although the 10-bit have a theoretical resolution of 1.2 ppb, in practice, they were unable to detect the first increase in concentrations (1.0 to 3.2 ppb; Figure 3, box 1). However, 10-bit sensors did respond to this initial change in concentration. All low-cost configurations continued to be responsive across the higher range of concentrations up to the maximum tested (305 ppb). Configuration F was able to detect all steps in concentration. The GGUN FL30 was unable to detect changes in concentrations greater than 200 ppb (Figure 3, box 2) due to saturation of the optical detector.

### 3.2. Sensitivity of Low-Cost Fluorometers to Temperature and Turbidity

#### 3.2.1. Thermal Sensitivity

**Figure 4:**
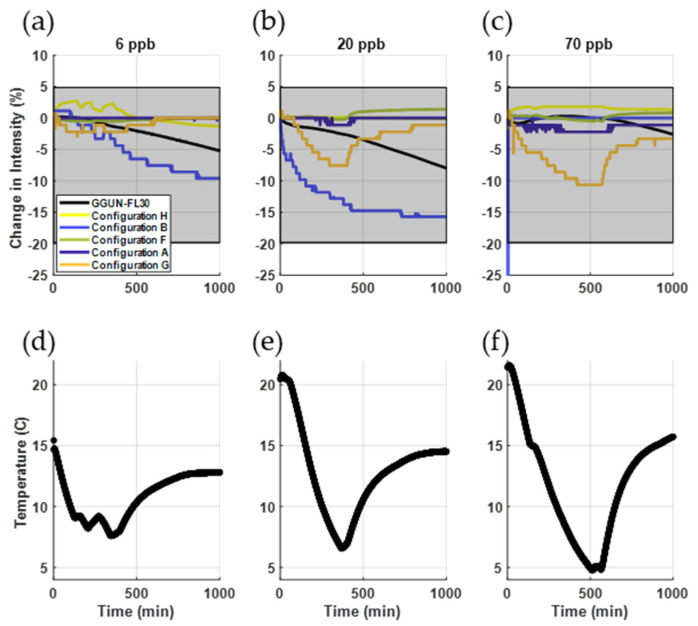
(**a**–**c**) Temperature dependence of uranine across the low-cost sensors and a commercial unit for three uranine concentrations. Shaded grey area represents the maximum and minimum change in intensity measured across GGUN FL30 units from our study and prior assessment of the same units [10,41]. (**d**–**f**) Temperature timeseries for Experiment 3.

Thermal sensitivity of the low-cost sensors was comparable to the commercial unit. While temperature sensors recorded similar trends in temperature (r^2^ = 0.97), the low-cost sensor recorded an overall smaller range in temperatures (15 to 18 °C) compared to the commercial sensor (5 to 21.5 °C). We attribute the divergence to the difference between sensor place (in the water for low-cost configurations; isolated from the water in the commercial fluorometer). The temperature dependence of fluorometers has been observed to vary with equipment, even across individual units of the same make and model [10,41]. The GGUN FL30 used in this study showed minimal temperature dependence, within the range of dependencies expected across GGUN units (Figure 4a–c). For comparison, Blaen et al. [10] found a change in intensities for uranine varied up to 15% across several GGUN units tested using the same testing protocol. The greatest variability, calculated as the percent change from the initial signal at room temperature, calculated from the low-cost sensors was 17%, measured with configuration B. Configuration H was most stable, with a maximum change in intensity of 3%. Temperature dependence was also observed in the logarithmic light sensors, while photoresistors were generally insensitive to temperature. The r^2^ for configurations were 0.38, 0.17, 0.56, 0.74, and 0.45 for the configurations A, B, F, G, H, respectively. The average r^2^ for the GGUN was 0.43. Overall, we find that sensitivity to temperature for some configurations (B and G) was greater than the particular commercial unit we tested, but within the range of expected sensitivities reported for these units in the literature [10].

#### 3.2.2. Turbidity Measurements and Interference with Fluorometry

**Figure 5:**
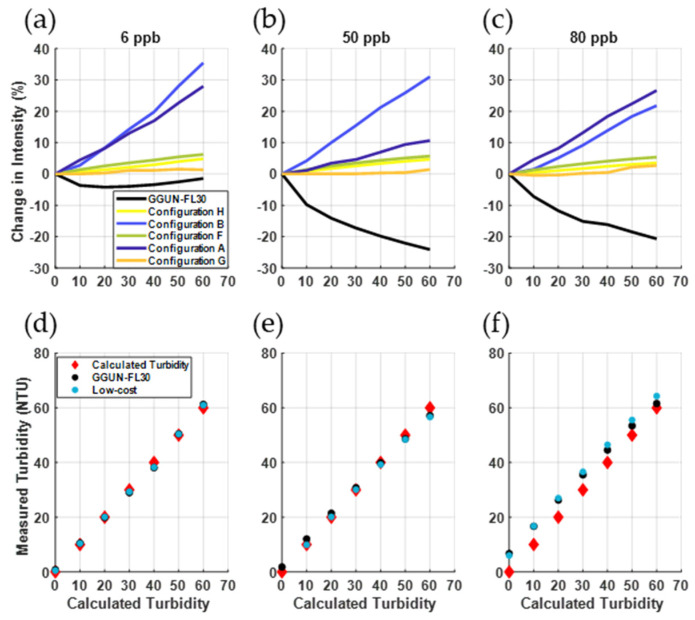
(**a**–**c**) Performance of the low-cost sensors at measuring turbidity. (**d**–**f**) Low-cost sensors were comparable to the commercial unit (r^2^ = 1.0). Sensors A and B showed the greatest sensitivity to changes in turbidity, comparable or greater than the commercial unit. Three of the five configurations were insensitive (<10% change in intensity) to changes to turbidity (middle column).

Turbidity measurements across all low-cost sensors achieved an r^2^ of 1.0 with the commercial unit (Figure 5), indicating a strong linear relationship between known turbidity and detected light transmission. All sensors reliably measured turbidity with varying uranine concentrations (Figure 5d–f) with r^2^ ranging from 0.76 to 1.0 for all concentration and turbidity configurations. The commercial unit showed fluorescence interference with turbidity similar to those documented in past studies (Figure 5a–c) [10]. Interference is particularly notable when both uranine concentration and turbidity are high (Figure 5c,f). The greatest decrease in intensity attributable to turbidity was 24% for the GGUN and 35% for the low-cost sensors (configuration B). However, uranine detection for three of the five low-cost sensors tested were insensitive to turbidity (<10% change in intensity for configurations F, G, H) while configurations A and B exhibited changes in intensity varying from 10–35%. Overall, changes in intensity due to turbidity for the low-cost configurations (1 to 35%) were comparable in magnitude to that observed for the GGUN FL30 tested here (−24 to −4%) and those reported by Blaen et al., [10] (−10% to 1%). The largest difference between the low-cost sensors and the commercial unit is the proportionality to turbidity. The changes in intensity for low-cost sensors were directly proportional to turbidity, while the particular commercial unit we tested showed an inverse relationship with turbidity.

## 4. Discussion & Conclusions

### 4.1. Low-Cost Fluorometers Can Achieve Fluorescent Tracer Sensitivity, Performing Comparably to Commercial Units across Operational Ranges for Solute Tracer Studies

The goal of this study was to prototype a capable, low-cost, single tracer fluorometer for a fraction of the price for a commercial unit. To that end, the configurations explored in this study demonstrate that a low-cost fluorometer can produce comparable data to a commercial unit–including performance across variability in turbidity and temperature–for a total cost of USD 59.13 for the components (Configuration H). Added cost would be associated with packaging in a configuration to meet applications (e.g., waterproof flow-through housing for stream solute tracers; cuvette holder for laboratory or grab sample analysis). Based on the performance across variable uranine concentrations, temperature, and turbidities, we recommend design configuration H. Comparison to the commercial unit resulted in a maximum r^2^ of 1.0 for concentrations of uranine, and the low-cost fluorometer configurations were sensitive at concentrations beyond the upper limit of the commercial unit. The fluorometer produced, here, obtained a functional resolution of 0.1 ppb and a working concentration range of 0.1 to >300 ppb under laboratory conditions, with measurement standard deviation ranging from 0.1 to 26.6 ppb across the operational range tested [36].

The results of this study also document the influence of both temperature and turbidity on measured fluorescence intensity, both of which are known to be important in interpretation of data from commercial units [10]. Across all configurations, the low-cost sensors experienced comparable sensitivity to temperature and turbidity to the commercial units that have been documented in the literature. While the particular commercial unit used in this study showed minimal dependence with temperature, identical units have shown greater dependence in past tests [10]. The commercial unit showed a dependence of fluorescence on turbidity that was non-linear with uranine concentration. Low-cost components ranged from insensitive to temperature and turbidity to comparably as sensitive to as the commercial units used in this study and others [10].

For an individual tracer, the on-board 8 and 10-bit pin(s) of the Adafruit Feather m0 Adalogger are capable of data in good agreement with the commercial unit given sufficient averaging of measurements. Comparison across uranine, temperature, and turbidity produced r^2^ values of 1.0, 0.97, and 1.0 between the low-cost fluorometer and the commercial unit. Overall performance of the low-cost fluorometer compared well to the commercial unit. The low-cost fluorometer was able to outperform the commercial unit in two areas. First, the GGUN FL30 has a memory capacity of 2 gigabytes, while the low-cost fluorometer can initialize larger external memory cards (32 gigabytes used in low-cost fluorometer) that may be useful in extended deployment. Next, the low-cost fluorometer was able to accurately measure concentrations above 300 ppb, while that saturated the optical sensors of the commercial unit. While this study did not explore the upper limit of either unit, the commercial unit was unable to accurately measure the 305 ppb concentration, suggesting higher concentrations would not perform well.

### 4.2. Low-Cost Fluorometers Are Readily Customized for Different Tracers and Could Be Adapted for Multi-Tracer Applications

Expansion of the designs tested beyond single tracers can also be accomplished with minimal additional costs. For instance, a configuration similar to the GGUN FL-30, consisting of a temperature probe, four excitation sources (630 nm, 570 nm, 470 nm, 525 nm), and four photodetectors would cost no more than 5% of a commercial unit depending upon exact selection of components and housings. Construction and programming of this low-cost fluorometer requires only rudimentary knowledge of electronics and coding due to the availability of open-source instructions and examples. Additionally, the low cost of parts and numerous locations to source components allow for rapid and affordable repairs/upgrades.

Additionally, placement of the 10 kΩ Precision Epoxy Thermistor changes the significance of changes in temperature. The observed variability in the low-cost fluorometer’s and GGUN’s temperature ranges, as a result of probe placement, indicate that, while both are measuring temperature, one is measuring sensor temperature (GGUN) and one is measuring environmental temperature (low-cost fluorometer). Measuring internal housing temperature is useful to temperature-correct component variability. However, these measurements have almost exclusively been used as a direct analog to system temperatures. Directly measuring water temperatures is the best placement to account for changes in fluorescence as a result of water temperature. At the cost of USD 4.00 per 10 kΩ Precision Epoxy Thermistor, measuring both component (in waterproof housing) and system (water) temperature would allow for variability in sensors and fluorescence to be divorced from each other.

**Figure 6:**
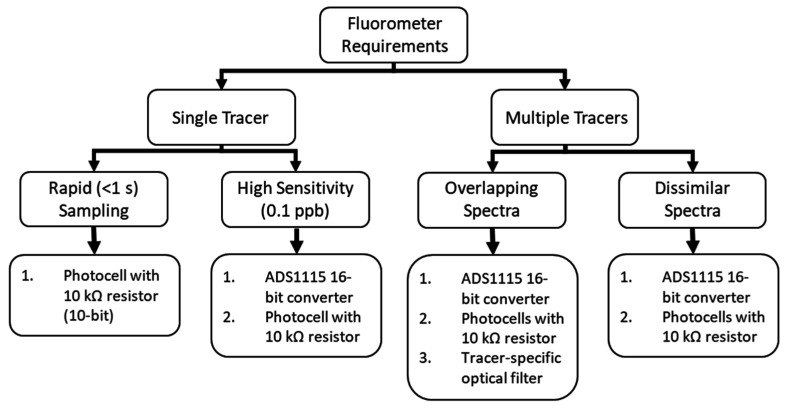
Flow chart of suggested component selection based upon application. Each design consists of the Adafruit Feather m0 Adalogger and DS3231 Precision Real Time Clock mounted together on the FeatherWing Doubler. Temperature (8-bit), turbidity (8-bit), and rapid sampling for individual tracers (10-bit) are made using the on-board pins of the Adafruit Feather m0 Adalogger.

To aid in sensor selection for future applications, we developed a flow chart based on fluorometer requirements (Figure 6). Designs using the 16-bit adaptor are capable of supporting up to 4 sensors per board, and 4 boards per controller, yielding a maximum of 16 sensors that are 16-bit resolution on a single controller. Notably, this many parallel operations would limit temporal resolution on any individual channel, but could be of interest for high resolution measurements made in close proximity, such as multiple depths on a minipoint sampler [42]. Optical filters, while improving r^2^ values, were found to not be cost effective for single tracers. However, multi-tracers applications may benefit from filters on the sources and/or detectors to minimize interference if simultaneous measurements are to be made.

## Figures and Tables

**Table 1:** Summary of commercial fluorometer cost and performance for uranine.

Commercial Unit	2020 Price (USD)	Manufacturer’s Stated Sensitivity for Uranine	Intended Application
Hach DR3900	7779	2 ppb	Spectrofluorometer
Hach DR900	1491	10 ppb	Field colorimeter
AquaLog	38,950	<1 ppb	Spectrometer
GGUN FL 30	8000	0.02 ppb	Field fluorometer
Trilogy Laboratory Fluorometer with Uranine Module	6865	0.01 ppb	Laboratory fluorometer
Cyclops-7F (Uranine Optics)	1865	0.01 ppb	Field fluorometer
Cyclops-6K (Chlorophyll Optics)	6375	0.25 ppb	Field fluorometer

**Table 2:** Fluorometer configurations tested in this study. Each configuration is shown along with the cost for unique components for that configuration, bit rate assessed, r^2^ with respect to the commercial fluorometer, and measurement uncertainty before averaging. Variants with 16-bit recording include an ADS1115 resolution converter. The total price includes the unique components plus the board, SD card, light sources (single tracer and turbidity), and one 10 kΩ Precision Epoxy Thermistor common to all configurations (USD 58.18).

Config. ID	Unique Hardware	Bit	r^2^	Range of Measurement Uncertainty ^a^	Unique Component Price (USD)	Total Price (USD)
A	Photocell w/1 kΩ resistor	10	0.95	0.0–90%	$0.95	$59.13
B	Photocell w/10 kΩ resistor	10	>0.999	0.0–54%	$0.95	$59.13
C	Photocell w/100 kΩ resistor	8	0.99	0.0–119%	$0.95	$59.13
D	Analog Light Sensor (ALS-PT19)	8	0.35	0.0–127%	$2.50	$60.68
E	High Dynamic Range Digital Light Sensor (TSL2591)	16	0.99	0.6–154%	$6.95	$65.13
F	Log-scale Analog Light Sensor (GA1A12S202)	16	0.92	−317–2860%	$3.95	$62.13
G	Log-scale Analog Light Sensor (GA1A12S202) w/optical filter ^b^	8	0.85	−1897–1558%	$80.95 ^c^	$139.13
H	Photocell w/10 kΩ resistor	16	0.99	0.3–53%	$0.95	$59.13
I	Log-scale Analog Light Sensor (GA1A12S202)	8	0.37	0.0–157%	$3.95	$62.13

^a^ For configurations A–I, we report the range of 95% confidence intervals (as 2σ/μ × 100%) for the 14 concentrations tested in Experiment 2. Uncertainty for each concentration is available on HydroShare [36]; ^b^ 1 cm^2^, 510–520 nm notch filter from PIXELTEQ; ^c^ price reflects the USD 3.95 sensor costs (identical to Configurations F and I) plus USD 77.00 for the optical filter.

## Data Availability

All data from this study are available in [36].

## References

[1] Höhne A., Mellerowicz K., Lischtschenko O., Lewandowski J. (2021). A novel device for in situ point measurements of fluorescent tracers in sediment pore water. Adv. Water Resour..

[2] Runkel R.L. (2015). On the use of rhodamine WT for the chatrcterization of stream hydrodynamics and transient storage. Water Resour. Res..

[3] Stream Solute Workshop (1990). Concepts and Methods for Assessing Solute Dynamics in Stream Ecosystems. J. N. Am. Benthol. Soc..

[4] Kvittingen E.V., Kvittingen L., Melø T.B., Sjursnes B.J., Verley R. (2017). Demonstrating basic properties of spectroscopy using a self-constructed combined fluorimeter and UV-Photometer. J. Chem. Educ..

[5] Bencala K.E., Rathbun R.E., Jackman A.P., Kennedy V.C., Zellweger G.W., Avanzino R.J. (1983). Rhodamine Wt Dye Losses in a Mountain Stream Environment. JAWRA J. Am. Water Resour. Assoc..

[6] Jankowski K., Schindler D.E., Lisi P.J. (2014). Temperature sensitivity of community respiration rates in streams is associated with watershed geomorphic features. Ecology.

[7] Salafi T., Zeming K.K., Lim J.W., Raman R., Seah A.W.R., Tan M.P., Zhang Y. (2019). Portable Smartphone-Based Platform for Real-Time Particle Detection in Microfluidics. Adv. Mater. Technol..

[8] Fischer H.B., List E.J., Koh R.C.Y., Imberger J., Brooks N.H. (1979). Mixing in Inland and Coastal Waters.

[9] Chung S.W., Gu R.R. (2003). Estimating time-variable transformation rate of atrazine in a reservoir. Adv. Environ. Res..

[10] Blaen P.J., Brekenfeld N., Comer-Warner S., Krause S. (2017). Multitracer Field Fluorometry: Accounting for Temperature and Turbidity Variability during Stream Tracer Tests. Water Resour. Res..

[11] Zepp R.G., Cline D.M. (1977). Rates of Direct Photolysis in Aquatic Environment. Environ. Sci. Technol..

[12] Debell T., Goertzen L., Larson L., Selbie W., Selker J., Udell C. (2019). OPEnS Hub: Real-Time Data Logging, Connecting Field Sensors to Google Sheets. Front. Earth Sci..

[13] Gillett D., Marchiori A. (2019). A low-cost continuous turbidity monitor. Sensors.

[14] Hossain A., Canning J., Ast S., Rutledge P.J., Li Yen T., Jamalipour A. (2015). Lab-in-a-Phone: Smartphone-Based Portable Fluorometer for pH Measurements of Environmental Water. IEEE Sens. J..

[15] Kelley C.D., Krolick A., Brunner L., Burklund A., Kahn D., Ball W.P., Weber-Shirk M. (2014). An affordable open-source turbidimeter. Sensors.

[16] Leeuw T., Boss E.S., Wright D.L. (2013). In situ measurements of phytoplankton fluorescence using low cost electronics. Sensors.

[17] Liu T., Wang W., Ding H., Yi D. (2019). Smartphone-Based Hand-Held Optical Fiber Fluorescence Sensor for On-Site pH Detection. IEEE Sens. J..

[18] Porter L.A., Chapman C.A., Alaniz J.A. (2017). Simple and inexpensive 3D printed filter fluorometer designs: User-friendly instrument models for laboratory learning and outreach activities. J. Chem. Educ..

[19] Wigton B.T., Chohan B.S., McDonald C., Johnson M., Schunk D., Kreuter R., Sykes D. (2011). A portable, low-cost, LED fluorimeter for middle school, high school, and undergraduate chemistry labs. J. Chem. Educ..

[20] Wigton B.T., Chohan B.S., Kreuter R., Sykes D. (2011). The characterization of an easy-to-operate inexpensive student-built fluorimeter. J. Chem. Educ..

[21] Ali A.S., Zanzinger Z., Debose D., Stephens B. (2016). Open Source Building Science Sensors (OSBSS): A low-cost Arduino-based platform for long-term indoor environmental data collection. Build. Environ..

[22] Laidlaw I.M.S., Smart P.L. (1977). An Evaluation of Some Fluorescent Dyes for Water \t\t\t Tracing. Water Resour. Res..

[23] Steppuhn H., Meiman J.R., Goodell B.C. (1971). Automatic detection of water-borne fluorescent tracers. Int. Assoc. Sci. Hydrol. Bull..

[24] Smart P.L., Finlayson B.L., Rylands W.D., Ball C.M. (1976). The relation of fluorescence to dissolved organic carbon in surface waters. Water Res..

[25] Lorenzen C.J. (1966). A method for the continuous measurement of in vivo chlorophyll concentration. Deep Res..

[26] Schreiber U., Schliwa U., Bilger W. (1986). Continuous recording of photochemical and non-photochemical chlorophyll fluorescence quenching with a new type of modulation fluorometer. Photosynth. Res..

[27] Schnegg P., Kennedy K. A new borehole fluorometer for double tracer tests. Proceedings of the Mass Transport in Fractured Aquifers and Aquitards.

[28] Jeong H., Shin S., Hwang J., Kim Y.J., Choi S. (2021). Open-Source Fluorescence Spectrometer for Noncontact Scientific Research and Education. J. Chem. Educ..

[29] Cucci C., Mignani A.G., Dall’Asta C., Pela R., Dossena A. (2007). A portable fluorometer for the rapid screening of M1 aflatoxin. Sens. Actuators B Chem..

[30] Tseng Y.C., Chu S.W. (2017). High spatio-temporal-resolution detection of chlorophyll fluorescence dynamics from a single chloroplast with confocal imaging fluorometer. Plant Methods.

[31] Bullis R., Coker J., Belding J., De Groodt A., Mitchell D.W., Velazquez N., Bell A., Hall J., Gunderson W.A., Gunderson J.E.C. (2021). The Fluorino: A Low-Cost, Arduino-Controlled Fluorometer. J. Chem. Educ..

[32] Bates H., Zavafer A., Szabó M., Ralph P.J. (2019). A guide to Open-JIP, a low-cost open-source chlorophyll fluorometer. Photosynth. Res..

[33] Khosravani M.R., Reinicke T. (2020). 3D-printed sensors: Current progress and future challenges. Sens. Actuators A Phys..

[34] Pascua J.A.A., Prado A.J.A., Solis B.R.B., Cid-andres A.P., Cambiador C.J.B. (2019). Trends in fabrication, data gathering, validation, and application of molecular fluorometer and spectrofluoromete. Spectrochim. Acta Part A Mol. Biomol. Spectrosc..

[35] Schnegg P.A. (2002). An inexpensive field fluorometer for hydrogeological tracer tests with three tracers and turbidity measurement. Groundw. Hum. Dev..

[36] Hixson J., Ward A.S. (2021). Supporting Data for Hixson and Ward, Hardware Selection and Performance of Low-Cost Fluorometers, HydroShare. https://www.hydroshare.org/resource/7e89f4916e84454e84d2000e61da6b47.

[37] Lemke D., Schnegg P.A., Schwientek M., Osenbrück K., Cirpka O.A. (2013). On-line fluorometry of multiple reactive and conservative tracers in streams. Environ. Earth Sci..

[38] Flett V., Maurice L., Finlayson A., Black A.R., MacDonald A.M., Everest J., Kirkbride M.P. (2017). Meltwater flow through a rapidly deglaciating glacier and foreland catchment system: Virkisjökull, Se Iceland. Hydrol. Res..

[39] Flury M., Wai N.N. (2003). Dyes as tracers for vadose zone hydrology. Rev. Geophys..

[40] Khamis K., Sorensen J.P.R., Bradley C., Hannah D.M., Lapworth D.J., Stevens R. (2015). In situ tryptophan-like fluorometers: Assessing turbidity and temperature effects for freshwater applications. Environ. Sci. Process. Impacts.

[41] Leibundgut C., Maloszewski P., Külls C. (2009). Tracers in Hydrology.

[42] Duff J.H., Murphy F., Fuller C.C., Triska F.J. (1998). A mini drivepoint sampler for measuring pore water solute concentrations in the hyporheic zone of sand-bottom streams. Limnol. Oceanogr..

